# Aging Rewires Neuronal Metabolism, Exacerbating Cell Death After Ischemic Stroke: A Hidden Reason for the Failure of Neuroprotection

**DOI:** 10.3390/ijms27010081

**Published:** 2025-12-21

**Authors:** Matvey Vadyukhin, Vladimir Shchekin, Petr Shegai, Andrey Kaprin, Grigory Demyashkin

**Affiliations:** 1 Department of Digital Oncomorphology, National Medical Research Centre of Radiology, 2nd Botkinsky Pass., 3, 125284 Moscow, Russia; 2 Institute of Translational Medicine and Biotechnology, I.M. Sechenov First Moscow State Medical University (Sechenov University), Trubetskaya st., 8/2, 119048 Moscow, Russia; 3 Research and Educational Resource Center for Immunophenotyping, Digital Spatial Profiling and Ultrastructural Analysis Innovative Technologies, Peoples’ Friendship University of Russia (RUDN University), Miklukho-Maklaya st., 6, 117198 Moscow, Russia; 4 Department of Urology and Operative Nephrology, Peoples’ Friendship University of Russia (RUDN University), Miklukho-Maklaya st. 6, 117198 Moscow, Russia

**Keywords:** ischemic stroke, aging, penumbra, apoptosis, neuroprotection

## Abstract

Aging profoundly modifies neuronal responses to ischemia. We aimed to define age-dependent features of neuronal metabolism and cell death after ischemic stroke by assessing NeuN, NSE, and Caspase-3 in human cortical neurons and by comparing transcriptional activity within PI3K/Akt/mTOR and PI3K/Akt/FOXO3a pathways across age groups. The aim of this study was to determine age-dependent features of neuronal metabolism and cellular degradation in ischemic stroke based on immunohistochemical assessment of NeuN, NSE, and Caspase-3 markers in human cerebral cortex neurons, as well as to conduct a comparative analysis of gene expression in the PI3K/Akt/mTOR and PI3K/Akt/FOXO3a signaling pathways involved in the regulation of neuronal survival and apoptosis. For the investigation, frontal cortex autopsies from patients with ischemic stroke (*n* = 154; “young”, “middle” and “elderly”; death ≤7 days post-onset) were examined. Histology (hematoxylin–eosin) and Nissl staining were used for morphology and neuron counts. Multiplex immunofluorescence (NeuN, NSE, Caspase-3) quantified metabolically active and apoptotic neurons, and the percentage of Caspase-3^+^ among NeuN^+^ cells was calculated. qRT-PCR measured PIK3CA, AKT2, MTOR, and FOXO3A expression in the infarct border zone. Based on our results, neuronal density and NeuN/NSE expression declined with aging, and the fraction of Caspase-3^+^ among NeuN^+^ neurons in the penumbra rose (young 42%, middle 82%, elderly 89%). Morphologically “intact” penumbral neurons frequently lacked NeuN/NSE, revealing covert dysfunction. Young brains showed balanced activation of PI3K/Akt/mTOR and PI3K/Akt/FOXO3a, whereas elderly brains exhibited reduced Akt/mTOR activity with FOXO3A predominance, consistent with pro-apoptotic, inflammatory, and dysregulated autophagic signaling. Thus, aging markedly reduces neuronal metabolic activity and increases apoptotic death in the infarct border zone after ischemic stroke. In older patients, there is an almost complete loss of NeuN and NSE expression in penumbral neurons with robust activation of the caspase cascade, whereas younger patients retain a pool of metabolically active neurons. Age-dependent dysregulation of PI3K/Akt signaling—characterized by FOXO3a hyperactivation and mTOR suppression—further promotes apoptosis and dysregulated autophagy. These changes likely underlie the limited efficacy of standard neuroprotection in ischemic stroke and support the need for age-tailored neurotropic therapy aimed at enhancing pro-survival pathways within the infarct border zone.

## 1. Introduction

Ischemic stroke (IS) remains one of the leading causes of disability and mortality among adults worldwide, particularly in the elderly population [1,2]. Morphological and molecular alterations in the brain following IS are characterized by extensive neuronal loss, disruption of energy metabolism, and activation of intracellular signaling pathways that regulate cell survival. Despite significant advances in neuroimaging and molecular-genetic techniques, the assessment of structural and metabolic disturbances in brain tissue still requires precise histochemical and immunohistochemical methods. These approaches not only verify neuronal death but also allow quantitative evaluation of metabolic impairment and apoptosis at the cellular level. To investigate age-associated changes in neuronal metabolism and cell degradation using advanced immunohistochemical techniques, we selected specific markers—Neuron-specific enolase (NSE, γγ-enolase) and Neuronal nuclear antigen (NeuN, also known as RBFOX3 or Fox-3)—while neuronal death was assessed by the expression of caspase-3, the terminal effector of the apoptotic cascade, in combination with analysis of key regulatory genes involved in the Phosphoinositide 3-kinase/AKT serine/threonine kinase 1/Mechanistic target of rapamycin (PI3K/Akt/mTOR) and Forkhead box O3a (PI3K/Akt/FOXO3a) signaling pathways.

NSE is a cytosolic glycolytic enzyme encoded by the *ENO2* gene that catalyzes the conversion of 2-phosphoglycerate to phosphoenolpyruvate at the ninth step of glycolysis. NSE is a highly specific marker of mature neurons and neuroendocrine cells, playing a critical role in Adenosine Triphosphate (ATP) generation required for maintaining action potentials and synaptic transmission [3]. Its distribution reflects the intensity of neuronal energy metabolism, and the degree of NSE immunoreactivity correlates with the preservation of neuronal functional activity. Studies have shown that decreased NSE expression in the cortex and hippocampus during neurodegenerative disorders coincides with synaptic loss, whereas elevated NSE levels in cerebrospinal fluid are considered a biochemical marker of neuronal injury [4,5]. In ischemic stroke, NSE exhibits a characteristic temporal pattern: rapid degradation in the necrotic core (0–6 h), subsequent decline in staining intensity within the infarct nucleus with focal enhancement in the penumbral zone (24–48 h), and partial recovery of expression by days 3–10 [6,7]. Thus, NSE serves as an informative indicator of neuronal functional integrity and reflects metabolic activity at various stages of ischemic injury.

Another important marker of mature neurons is NeuN, a nuclear-cytoplasmic protein belonging to the Fox family that participates in the regulation of alternative pre-mRNA splicing and the expression of genes associated with neuronal differentiation and synaptic plasticity [8,9]. Under normal conditions, NeuN is predominantly localized within the nucleus and interchromatin granules of neurons; its expression begins at the final stage of neurogenesis and persists throughout the neuron’s lifespan. A decrease in NeuN immunoreactivity observed in neurodegenerative diseases, epilepsy, and ischemic injury reflects the loss of mature neurons and disturbances in post-transcriptional regulation [10,11]. During cerebral ischemia, NeuN frequently loses its nuclear localization and translocates into the cytoplasm or dendrites, a process linked to calpain-dependent proteolysis of RBFOX3 and disruption of nucleocytoplasmic transport—an early indicator of neuronal stress dysfunction that precedes cell death.

Moreover, ischemic stroke rapidly initiates energy deficiency, oxidative stress, inflammation, and apoptosis—key processes driving neuronal death [12]. However, the role of major cellular response regulators, such as the PI3K/Akt/mTOR and PI3K/Akt/FOXO3a signaling pathways, which control autophagy, inflammation, cell survival, and apoptosis, remains insufficiently explored despite extensive evidence of their involvement [13,14]. Limited studies have demonstrated that activation of PI3K/Akt may exert neuroprotective effects by reducing infarct volume and neuronal loss [15,16]. The underlying mechanism involves phosphorylation of Akt at Threonine308 and Serine473, leading to inactivation of pro-apoptotic proteins (Glycogen Synthase Kinase-3 Beta, Bcl2-associated agonist of cell death, Apoptosis signal-regulating kinase 1, cellular tumor antigen p53) and activation of Mechanistic Target of Rapamycin Complex 1 (mTORC1), a key regulator of protein synthesis and autophagy [17]. In contrast, the Akt/FOXO3a branch induces the expression of pro-apoptotic genes such as *BIM* (BCL2-like 11) and *FasL* (Fas Ligand), and its dysregulation may promote inflammation and neuronal death [18]. It has been proposed that PI3K/Akt activity declines with age due to attenuation of the Insulin-like Growth Factor 1 (IGF-1) signaling cascade and autophagic dysfunction [19], whereas hyperactivation of FOXO3a under conditions of Akt deficiency triggers pro-apoptotic signaling [20].

Thus, comprehensive immunohistochemical assessment of the markers NSE and NeuN enables evaluation of the metabolic activity of penumbral neurons, while analysis of caspase-3 expression—the terminal effector of apoptosis—together with activation of the PI3K/Akt/mTOR and PI3K/Akt/FOXO3a signaling pathways allows not only clarification of the morphological boundaries of neuronal degeneration but also identification of the molecular mechanisms underlying the age-dependent imbalance between neuronal survival and death in stroke. The obtained findings may serve as a foundation for developing age-specific strategies for neuroprotective therapy. The revealed integrative patterns contribute to a deeper understanding of the pathogenesis of ischemic stroke in the context of cortical neuronal aging.

The aim of the study was to identify age-dependent features of neuronal metabolism and cellular degradation in ischemic stroke based on immunohistochemical assessment of the markers NeuN, NSE, and Caspase-3 in human cortical neurons. A comparative analysis of gene expression within the PI3K/Akt/mTOR and PI3K/Akt/FOXO3a signaling pathways, which regulate neuronal survival and apoptosis, was also conducted across different age groups.

## 2. Results

A total of 154 patients were enrolled, divided into three age-based cohorts: young (n = 38, mean age 34.8 ± 4.2 years), middle (n = 51, 51.1 ± 4.8 years), and elderly (n = 65, 69.3 ± 5.4 years). The sex distribution did not differ significantly across groups (*p* = 0.315). According to ICD-10 classification, the majority of patients were diagnosed with ischemic stroke (I63.3/I63.4), while the elderly cohort showed a significantly higher proportion of patients with ICD-10 code I67.8 (87.7% vs. 2.6% in the young group; *p* < 0.001). Based on SSS-TOAST etiological classification, the proportions of atherosclerotic and cardioembolic strokes were comparable across age groups (no significant intergroup differences). Time-to-death analysis revealed a statistically significant difference among groups (*p* = 0.007). In younger patients, death most frequently occurred on day 3, whereas in the elderly group a greater proportion of deaths occurred within the first two days post-stroke (*p* = 0.035), suggesting a more rapid and severe disease course in older patients (Supplementary Materials Table S1).

**Figure 1:**
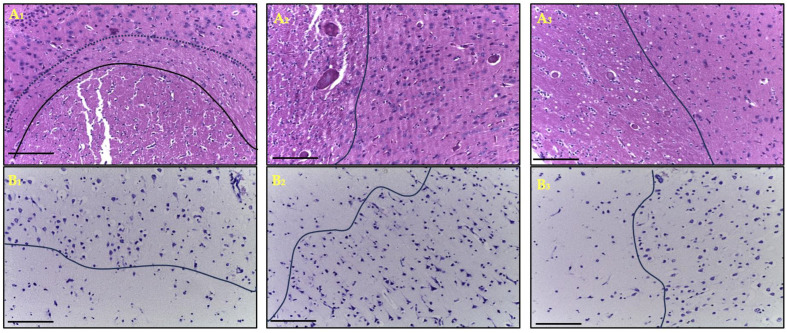
Frontal lobe of the cerebral cortex one day after ischemic stroke in the young (**A_1_**,**B_1_**), middle (**A_2_**,**B_2_**) and elderly (**A_3_**,**B_3_**) age subgroups: hematoxylin and eosin staining (**A_1_**–**A_3_**); Nissl staining (**B_1_**–**B_3_**), magn. ×200 (**A**). The micrographs show the infarction core: below the solid line (**A_1_**,**B_1_**) and to the left of the solid line (**A_2,3_**,**B_2,3_**). The dotted line (**A_1_**) outside the solid line delimits the area of delayed necrotic transformation of the penumbra. Above (**A_1_,B_1_**) or to the right (**A_2,3_,B_2,3_**) of the solid line is the penumbra zone with neurons in it. Scale bar—100 μm.

In the morphological examination of cortical brain samples from patients with ischemic stroke (n = 109), on the first day after onset, a morphological picture characteristic of infarction was observed, including a pannecrotic zone (infarct core) with a marked reduction in the number of neurons, cytoplasmic and perikaryonal eosinophilia, and nuclear pyknosis. Within the ischemic focus, pronounced microcirculatory disturbances were noted—venous hyperemia, perivascular edema (dilatation of Virchow–Robin spaces), erythrocyte aggregation, and capillary stasis. In the penumbra (named infarct border zone after 24 h), these alterations were less pronounced and were accompanied by moderate infiltration of lymphocytes and polymorphonuclear cells. Given that the penumbra is a dynamic and blood-flow-based concept and the evidence that this zone rarely persists longer than 24 h, we also use the term infarction border zone to refer to the area of damaged neurons outside the infarction core. In some fragments of the infarct border zone, glial cell hyperplasia (gliosis) was observed. The most severe morphological changes were recorded in elderly patients. At later time points, younger individuals exhibited signs of glial scar formation and a more distinct boundary between the infarct core and the infarct border zone. In contrast, in elderly patients, continued expansion of the infarct core was observed up to the seventh day, primarily due to the loss of cells within the infarct border zone (Figure 1).

In most examined cases (n = 127), the arterial walls exhibited signs of sclerosis, hyalinosis, and atrophy of smooth muscle cells, consistent with the cardioembolic type of ischemic stroke. In a number of cases (n = 76), secondary vascular alterations were observed, including lipoid and fibrotic changes, the presence of atherosclerotic plaques, hyalinosis, and thinning of the muscular layer, which are characteristic of the atherothrombotic variant of stroke. In the intact cortical regions, the typical histological architecture was preserved at all time points, with clearly distinguishable layers: molecular, external granular, pyramidal, internal granular, ganglionic, and multiform (polymorphic) cell layers, as well as visible elements of macroglia and microglia. At the same time, the number of neurons in elderly patients was significantly lower (by 19.2%) compared to that in young individuals (Table 1).

Multiplex immunohistochemical analysis of the cerebral cortex revealed a general age-related decrease in neuronal density. It is noteworthy that as early as the first day after stroke onset, the number of metabolically active (functionally intact) NeuN^+^NSE^+^ neurons in the infarct border zone were significantly lower than the number of structurally preserved neurons identified by histological examination (Table 2).

In ischemic stroke, no positively stained neurons were detected in the pannecrotic zone (infarct core), whereas in the intact cortical regions, moderate neuronal staining for NeuN and partial staining for NSE were observed, along with only a few Caspase 3^+^ neurons—mainly in the elderly group—at all time points examined.

**Figure 2:**
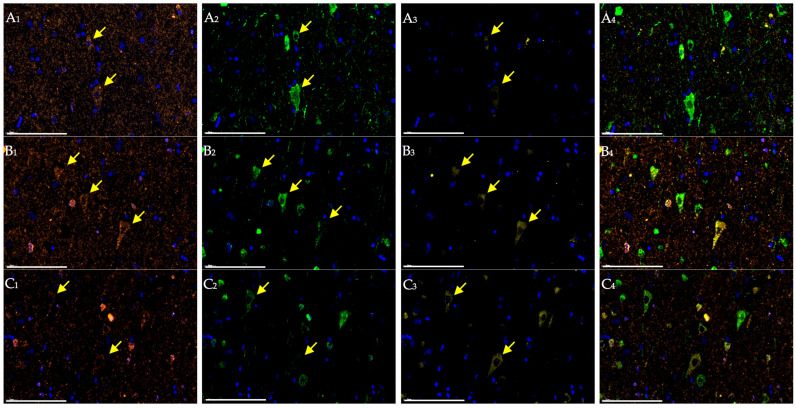
Cerebral cortex of patients one day after ischemic stroke in young (**A**), middle (**B**), and elderly (**C**) groups. Multiplex immunohistochemical staining with antibodies against NeuN (**A_1_**–**C_1_**; orange fluorescence; Opal 647), NSE (**A_2_**–**C_2_**; green fluorescence; Opal 488), and Caspase 3 (**A_3_**–**C_3_**; yellow fluorescence; Opal 540) as well as merged images (**A_4_**–**C_4_**); magn. ×400. Scale bar—100 μm. Images were acquired using the Vectra^®^ Polaris Automated Quantitative Pathology Imaging System (Akoya Biosciences, Marlborough, MA, USA). In the field of view: positively stained pyramidal neurons (yellow arrows).

Detailed analysis of cortical images from young patients with ischemic stroke revealed pyramidal neurons within the infarct border zone that retained normal cytological features and exhibited high NeuN and NSE staining intensity at early stages, presumably of compensatory nature (Figure 2). However, this was accompanied by activation of Caspase 3 expression—the terminal effector of the apoptotic cascade. Furthermore, this group demonstrated a reduction in NeuN expression in the nuclei and an abnormal diffuse redistribution of the protein within the cytoplasm, including dendritic processes. In middle patients, moderate alterations in NeuN and NSE expression were observed, along with more intense immunolabeling of neurons with antibodies to Caspase 3 (Figure 2).

**Figure 3:**
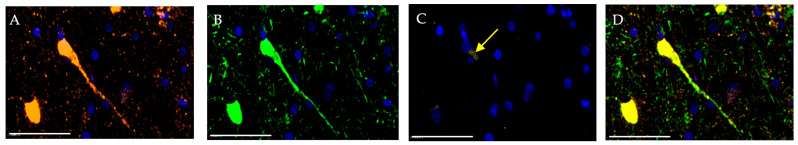
Cerebral cortex of a young patient one day after ischemic stroke. Multiplex immunohistochemical staining with antibodies against NeuN (**A**; orange fluorescence; Opal 647), NSE (**B**; green fluorescence; Opal 488), and Caspase 3 (**C**; yellow fluorescence; Opal 540) as well as merged image (**D**); magn. ×400. Images were acquired using the Vectra^®^ Polaris Automated Quantitative Pathology Imaging System (Akoya Biosciences, Marlborough, MA, USA). Scale bar—50 μm. The image shows a giant pyramidal neuron (NeuN^+^NSE^+^Caspase-3^+^) located at the boundary between the penumbral zone and intact tissue. High expression levels of NeuN and NSE are observed predominantly in the nucleus, Nissl substance, and dendrite, while termination of the apoptotic cascade (detected by Caspase 3 staining) is evident in the perikaryon (yellow arrow).

Particular attention should be given to the altered distribution of the examined markers in ischemic stroke. Upon closer evaluation, morphologically preserved giant pyramidal neurons were identified within the infarct border zone, in which NeuN was distributed not only in the nucleus but also in the cytoplasmic compartment—including Nissl substance and dendritic processes. In the same neurons, a high level of NSE expression was detected. However, during the first days after ischemic stroke, Caspase 3^+^ fluorescence appeared, indicating early activation of the apoptotic cascade, predominantly within the perikaryon (Figure 3).

As early as the first day in elderly patients, NeuN^+^ neurons were almost absent in the penumbra, and NSE expression was markedly reduced compared with that in young individuals. This was accompanied by a pronounced increase in Caspase 3 immunolabeling in most surviving neurons, confirming activation of the apoptotic cascade as one of the key mechanisms of cell death. Notably, despite these molecular alterations, the affected neurons retained a visually normal morphology for a prolonged period. At the same time, NSE expression displayed not only a neuronal pattern. Antibodies against NSE stained several small cells within areas of gliosis previously identified in the infarct border zone during morphological examination (Figure 2).

**Figure 4:**
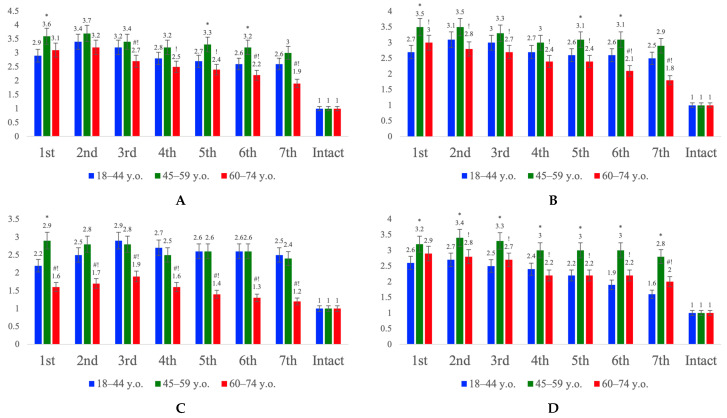
Relative mRNA expression of genes *PIK3CA* (**A**), *AKT2* (**B**), *MTOR* (**C**) and *FOXO3A* (**D**) in homogenized cortical tissue from patients with ischemic stroke in young (18–44 y.o.), middle (45–59 y.o.), and elderly (60–74 y.o.) groups, graph. Control values represent mRNA expression levels of the studied genes in the intact cortical zone for each age group. Intergroup differences were analyzed using the one-way ANOVA with Bonferroni adjustment when applicable using SPSS Statistics 12 for Windows (IBM Analytics, Armonk, NY, USA). Statistically significant differences: *—45–59 vs. 18–44; #—60–74 vs. 18–44; !—60–74 vs. 45–59; *p* < 0.05.

Analysis of the expression of key genes of the PI3K/Akt/mTOR and PI3K/Akt/FOXO3a signaling pathways in the cerebral cortex revealed pronounced age-related differences in their activation following ischemic stroke (Figure 4). In young patients, a balanced activation of both cascades was observed: expression of *PIK3CA*, *AKT2*, *MTOR*, and *FOXO3A* increased during the early period and then gradually declined to moderate levels, reflecting the preservation of regulatory mechanisms and reversible activation of neuroprotective and autophagic processes. In the middle group, a moderate increase in the expression of all markers was detected, with a tendency toward stronger transcriptional activation of *FOXO3A*, indicating the onset of an age-related imbalance between the PI3K/Akt signaling branches. In the elderly group, there was a general decrease in the transcriptional activity of all PI3K/Akt components, while *FOXO3A* expression levels significantly exceeded those of *MTOR*, suggesting a regulatory shift toward hyperactivation of the PI3K/Akt/FOXO3a axis. This redistribution likely reflects age-associated attenuation of pro-survival mechanisms, accompanied by a predominance of autophagic, pro-apoptotic, and proinflammatory effects resulting from dysregulated PI3K/Akt/FOXO3a signaling, which may contribute to increased neuronal vulnerability to ischemic injury in elderly patients.

## 3. Discussion

The present study demonstrates clear age-dependent differences in cortical neuronal responses to ischemia. Elderly patients exhibited markedly reduced metabolic activity and almost universal activation of apoptotic pathways within the infarct border zone, which was consistent with the more severe and rapidly progressing clinical course reported previously. These findings suggest that age-related structural and molecular alterations of the cerebral cortex predispose older individuals to accelerated neuronal injury and may limit the efficacy of standard neuroprotective strategies due to pronounced biological heterogeneity. Our study was designed to investigate age-dependent molecular and cellular patterns, and we applied strict inclusion and exclusion criteria to avoid confounding factors such as neurodegenerative, infectious, oncologic, or systemic inflammatory conditions, which are highly prevalent in individuals over 70 years. As a result, obtaining suitable autopsy material from the oldest age category was extremely challenging, which explains the absence of patients above 74 years in our cohort. The male predominance reflects the actual sex distribution of autopsies meeting all inclusion criteria during the study period rather than an intentional selection bias.

Multiplex immunohistochemistry provided higher sensitivity than routine histological staining, revealing a considerable population of morphologically preserved yet metabolically impaired neurons. Although these neurons appeared “intact” under Hematoxylin and Eosin as well as Nissl stains, multiplex labeling showed loss of NeuN and NSE expression together with early activation of Caspase 3, indicating covert injury consistent with earlier observations [21]. The presence of neurons with activated apoptotic cascades but without overt morphological degeneration aligns with the concept of delayed neuronal death and progressive expansion of the infarct core described previously [22,23,24]. This approach therefore enables more accurate detection of neuronal vulnerability in the infarct border zone and may serve as an important diagnostic tool for assessing the volume of secondary lesions.

### 3.1. Age-Related Modulation of Apoptotic Mechanisms in Penumbral Neurons

Multiplex immunohistochemical analysis revealed that as early as one day after stroke onset, a substantial proportion of neurons within the penumbra contained activated Caspase 3, the terminal effector of apoptosis. In the young and middle groups, the absolute number of Caspase 3^+^ neurons exceeded that observed in elderly patients. However, when the percentage of dying (caspase-positive) cells among the total number of neurons (NeuN^+^) in the penumbra was calculated, this value increased with age—42% in young patients versus 89% in elderly individuals. Thus, the apparent decrease in the absolute number of Caspase 3^+^ neurons in the elderly likely reflects reduced neuronal density rather than attenuation of apoptosis, which is consistent with the findings of other researchers [25]. Based on these observations, we developed a method for calculating the “Caspase^+^/NeuN^+^ neuron ratio,” which can serve as an applied diagnostic criterion for assessing apoptotic neuronal death in brain tissue samples following ischemic stroke. Although Caspase-3 is commonly used as an indicator of apoptotic signaling, its specificity in ischemic tissue is not absolute, and the absence of confocal imaging may theoretically allow minimal signal overlap. These factors should be considered as potential, though not definitive, limitations of our methodology.

Moreover, the high level of neuronal staining with anti-caspase 3 antibodies in the infarct border zone of elderly patients indicates that by the first day after stroke onset, the apoptotic cascade had already been activated in nearly all surviving cells. This represents a fundamental difference from younger patients, in whom a considerable proportion of penumbral neurons remain viable and can potentially be preserved with timely therapeutic intervention [26]. The obtained results partly explain the more severe clinical course of stroke in elderly patients [27]. In other words, their adaptive and compensatory potential is critically low, and the therapeutic window for effective intervention is extremely limited. In addition, in some cases we observed that in young patients, the infarct zone became demarcated early by glial scar formation, whereas in most elderly individuals, the infarct volume continued to expand progressively. This may account for the higher rate of late mortality observed in the elderly cohort [28]. Thus, the near-total activation of Caspase 3 in penumbral neurons of older patients underlies the rapid expansion of the infarct zone due to apoptotic death of neurons that were previously considered conditionally preserved.

### 3.2. Influence of Age on Neuronal Metabolic Activity

Co-localization of the markers NeuN and NSE selected in the present study provides insight not only into the number of neurons but also into their functional and metabolic activity [5,29]. The Neuronal nuclear antigen (NeuN, RBFOX3) is involved in the regulation of mRNA splicing and is normally present in the nuclei and perikaryon of nearly all differentiated neurons [30]. The intensity of NeuN immunolabeling correlates with neuronal integrity, whereas suppression of its expression indicates cellular distress [31]. Neuron-specific enolase (NSE), a key cytosolic enzyme of glycolysis, serves as an indicator of the neuronal energy metabolism level [32]. Multiplex analysis revealed that in young patients, large pyramidal neurons with high NeuN and NSE expression were still present in the penumbra during the first day after stroke onset. This finding indicates enhanced metabolic activity, reflecting compensatory hyperenergization in response to ischemia [33]. Indeed, cytologically preserved neurons within the infarct border zone must adapt to oxygen and glucose deficiency by shifting toward anaerobic glycolysis, which is confirmed by elevated NSE expression. However, paradoxically, some of these neurons were also positively stained for Caspase 3. It is likely that the transient increase in glycolytic activity does not prevent subsequent cell death but rather delays it, providing short-term metabolic support until the genetic program of apoptosis is executed.

In contrast, in the elderly group, most neurons exhibited low metabolic activity, which may be explained by the reduced compensatory and adaptive potential of brain structures [34]. Immunolabeling for NeuN and NSE in this group was markedly decreased, despite the apparent preservation of some cells under routine histological staining. NeuN deficiency may also indicate impaired protein-synthetic function of neurons, particularly in elderly patients, supporting the role of ontogenetic changes [35]. The decline in protein-synthetic capacity may be indirectly related to the fact that NeuN is associated with nuclear chromatin and Nissl substance, that is, with granular endoplasmic reticulum and ribosomes. Its loss likely reflects ribosomal destabilization and cessation of protein synthesis, further contributing to the energetic and trophic deficiency of the cells [36].

In addition, the present study revealed an interesting phenomenon of NeuN redistribution in neurons of the infarct border zone. We observed diffuse accumulation of this protein within the cytoplasm and even dendritic processes instead of the predominantly nuclear localization typical under normal conditions. Such abnormal NeuN distribution corresponds to isolated reports in the literature, where this pattern is associated with calpain-dependent proteolytic cleavage of RBFOX3 combined with disruption of nuclear envelope transport under ischemic conditions [37,38]. Our results suggest that the efflux of NeuN from the nucleus represents an early marker of neuronal functional failure preceding irreversible morphological alterations.

It is noteworthy that in the cerebral cortex of elderly patients, not only the expression of neuronal markers but also the nature of cellular responses within the infarct border zone was altered. In some fragments from this group, pronounced glial hyperplasia (gliosis) was observed along the periphery of the infarct, and several astrocytes and oligodendrocytes were positively stained with antibodies against NSE. Although NSE is traditionally regarded as a neuron-specific protein, it is known that hybrid isoforms of this enzyme (αγ-dimers) can be expressed by glial cells in the central nervous system [5]. The presence of NSE^+^ small cells in the infarct border zone of elderly patients is likely associated with translocation of this enzyme from damaged neuronal processes to macro- or microglia. Another possible explanation for this phenomenon is a metabolic shift in glial cells toward enhanced glycolysis under ischemic conditions [39]. However, this hypothesis requires further clarification in subsequent studies. Nevertheless, the analysis of NeuN and NSE co-localization clearly demonstrates the inability of neurons in older individuals to maintain adequate energy metabolism during stroke compared with younger patients. This finding supports the concept of age-associated decline in neuronal plasticity and the adaptive-compensatory capacity of the cerebral cortex.

### 3.3. Role of Dysregulation of the Akt/mTOR and Akt/FOXO3a Signaling Pathways

The present study revealed significant age-related differences in the activity of the PI3K/Akt/mTOR and PI3K/Akt/FOXO3a signaling pathways, which are responsible for regulating cellular survival and death [40,41]. Quantitative Reverse Transcription Polymerase Chain Reaction (qRT-PCR) analysis showed that in young patients, ischemic stroke was accompanied by coordinated activation of both the pro-survival PI3K/Akt/mTOR cascade and the stress-induced PI3K/Akt/FOXO3a axis [42]. Previous studies have reported both beneficial and detrimental effects of PI3K/Akt/FOXO3a activation, depending on the affected organ and the nature of the insult [20]. However, our findings suggest that this signaling pathway directly contributes to neuronal death within the infarct border zone.

In cortical homogenates from young patients, expression levels of Phosphatidylinositol-4,5-bisphosphate 3-kinase catalytic subunit alpha (*PIK3CA*), AKT serine/threonine kinase 2 (*AKT2)*, Mammalian Target of Rapamycin (*MTOR*), and Forkhead box O3a (*FOXO3A*) genes were found to increase almost equally during the first 24 h after stroke, followed by a trend toward normalization at later stages. This “balanced” response can be interpreted as the concurrent activation of mechanisms promoting cell survival (primarily through the Akt–mTOR pathway) and mechanisms aimed at damage resolution (via moderate activation of FOXO3a-dependent genes involved in autophagy and apoptosis). Notably, in the middle group, signs of dysregulation were observed: a relative increase in FOXO3A expression compared with MTOR indicated a shift in signaling balance toward a pro-apoptotic response.

Finally, in elderly patients, the overall transcriptional activity of the PI3K/Akt pathway components was markedly reduced, consistent with previous reports describing age-related suppression of the insulin-like growth factor 1 (IGF-1) signaling cascade and associated kinase pathways as potential underlying mechanisms [19,43]. Against this background, FOXO3A expression levels in our study were significantly higher than in young patients, whereas mTOR expression remained decreased. In other words, in the cerebral cortex of elderly individuals, ischemia induces a pathological predominance of the Akt/FOXO3a axis with relative insufficiency of Akt/mTOR signaling [44]. Considering that under normal physiological conditions Akt suppresses FOXO proteins, ischemic stroke may result in insufficient Akt (and consequently mTOR) activity, leading to uncontrolled FOXO3a activation [42]. This, in turn, triggers cell death, dysregulated autophagy, and the expression of proinflammatory interleukins. As a result, immune cell migration into the infarct border zone increases, promoting autoimmune neuronal injury and expansion of the infarct core. These findings are crucial for understanding the molecular mechanisms underlying neuronal death.

Under normal conditions, the presence of trophic factors such as IGF-1 activates the PI3K/Akt pathway, leading to suppression of FOXO3a expression: activated Akt phosphorylates FOXO3a, retaining it in the cytoplasm, while simultaneously initiating mTOR-mediated programs of protein synthesis, cellular growth, and survival [42]. It is likely that in older patients, insufficient Akt activity results in the accumulation of unphosphorylated FOXO3a, which translocates to the nucleus, where it functions as a transcription factor in the stress response. FOXO3a is known to regulate numerous genes associated with apoptosis and inflammation [45]. In particular, it induces the expression of pro-apoptotic proteins (Bim, FasL, Puma, and others), promotes the synthesis of proinflammatory cytokines, and, in some cases, enhances autophagy and ubiquitin–proteasome-mediated proteolysis [46,47].

In young patients, the effects of FOXO3a are partially counterbalanced by mTOR activity and other pro-survival proteins, such as the anti-apoptotic Akt/Bcl-2 axis. In contrast, the data obtained in the present study indicate that in older individuals, ischemia induces uncontrolled nuclear activation of FOXO3a accompanied by insufficient activity of compensatory survival pathways. The consequences of this include the following: enhanced apoptosis through activation of the caspase cascade; chronic inflammation due to hyperactive FOXO3a–mediated induction of proinflammatory cytokine production; and dysregulated autophagy, characterized by autophagic cell death against a background of suppressed mTOR signaling. Collectively, these processes lead to increased neuronal loss in the infarct border zone and progression of necrosis in the cerebral cortex.

Our findings are consistent with the results of previous studies. For instance, pharmacological activation of PI3K/Akt has been shown to reduce infarct volume in experimental stroke models, whereas inhibition of Akt exacerbates neuronal death [15,16]. There is also evidence that FOXO3a hyperactivation is associated with greater lesion volume due to the induction of uncontrolled autophagy, apoptosis, and proinflammatory cytokine synthesis during ischemia [48]. In the present study, this phenomenon was demonstrated for the first time in human brain tissue. The predominance of FOXO3A over MTOR expression in the infarct border zone may thus serve as a molecular marker of insufficient neuroprotective mechanisms in elderly patients. This observation is particularly important for the development of new potential targets for neuroprotective therapy. Modulation of the Akt/FOXO3a axis—such as targeted inhibition of FOXO3a or activation of Akt—may prove effective in older individuals, whereas such an approach may be less beneficial in younger patients. This finding underscores the importance of considering patient age when selecting neurotropic treatment strategies and may partially explain the limited efficacy of standard neuroprotective therapies in age-heterogeneous cohorts of patients with ischemic stroke [49,50].

### 3.4. Influence of Patient Age on Neuronal Death and Stroke Outcomes

**Figure 5:**
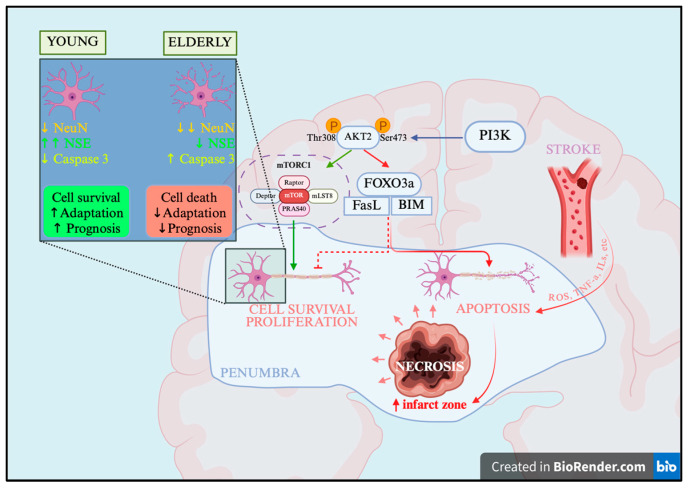
Mechanisms of pathogenesis and neuronal death after ischemic stroke, scheme. PI3K—Phosphatidylinositol 3-kinase, Akt2—Protein kinase B isoform 2, Thr—threonine, Ser—serine, FOXO3a—Forkhead box O3a, mTORC1—Mechanistic Target of Rapamycin Complex 1, FasL—Fas Ligand (CD95L), BIM—Bcl-2-interacting mediator of cell death. Created in BioRender.com, https://app.biorender.com/illustrations/68f73d20562823245973809b?slideId=fb2d556b-50e4-40fa-9ed7-fe7ce08ea583, accessed on 11 October 2025.

The results of the present study demonstrate significant age-associated alterations in the mechanisms of neuronal death following ischemic stroke. In young patients, neuronal injury in the cerebral cortex can be considered partially compensated: during the first 24 h, a pannecrotic zone forms, while a portion of penumbral neurons retains normal cytology and partial metabolic activity—a state often referred to as “neuronal lethargy” [21]. These neurons exhibit compensatory enhancement of metabolic activity, as evidenced by increased NeuN and NSE expression, together with moderate activation of the caspase cascade, suggesting a potential for reversibility. At the molecular level, penumbral neurons appear to maintain a partial balance between the PI3K/Akt/mTOR and PI3K/Akt/FOXO3a signaling pathways. Collectively, this enables temporary maintenance of protein synthesis, energy metabolism, and partial restriction of apoptosis in the infarct border zone (Figure 5), which may contribute to the preservation of cells in this region, partial prevention of infarct core expansion, and improved clinical outcomes.

In contrast, in elderly patients, by the first day after stroke onset, nearly complete loss of NeuN- and NSE-positive neurons was observed in the penumbra, accompanied by a sharp increase in the proportion of Caspase 3 expressing cells. This likely reflects reduced adaptive and metabolic capacity of neurons. The loss of NeuN indicates suppression of protein-synthetic activity, while decreased NSE reflects impairment of glycolytic metabolism. Molecular analysis revealed predominance of FOXO3A transcriptional activity combined with reduced expression of its suppressors, Akt and mTOR, suggesting dominance of pro-apoptotic signaling. Moreover, in this patient group, the mechanisms of autophagy, inflammation, and apoptosis lose regulation and become integral components of ischemic stroke pathogenesis, accelerating neuronal death in the infarct border zone and expansion of the infarct zone (Figure 5). This contributes to a more severe clinical course and poorer prognosis.

This set of findings is highly relevant to current efforts aimed at improving functional outcomes after ischemic stroke, particularly in the era of widespread recanalization therapies [51]. Recent Stroke Treatment Academic Industry Roundtable (STAIR) recommendations emphasize that successful reperfusion must be accompanied by effective cerebroprotective strategies capable of preserving the metabolic viability of penumbral neurons [52,53]. Our data suggest that age-dependent dysregulation of neuronal survival pathways (an imbalance between Akt/mTOR and Akt/FOXO3a) may largely explain why many neuroprotective agents fail to translate into clinical benefit in heterogeneous patient populations. These results highlight the importance of developing age-tailored cerebroprotective approaches that enhance pro-survival signaling and stabilize neuronal metabolism following recanalization, especially in older groups.

In summary, with advancing age, the cerebral cortex progressively loses its capacity for metabolic adaptation and cellular survival in response to ischemia. In the cortical tissue of elderly patients, nearly all neurons in the infarct border zone exhibited signs of apoptotic death at early stages, whereas in younger individuals, a population of cells with recovery potential was preserved. This difference reflects an age-associated decline in neuronal plasticity and energetic resilience, leading to rapid expansion of the necrotic focus and worse stroke outcomes. The findings suggest that age-dependent dysregulation of the Akt/mTOR and Akt/FOXO3a signaling pathways, in combination with impaired energy metabolism, underlies the observed differences in the severity and outcomes of ischemic stroke. These results further emphasize the need to develop neuroprotective approaches aimed at restoring the regulatory function of these signaling pathways and enhancing neuronal metabolic stability in elderly patients. The data also indicate that such neuroprotective strategies may be less effective in younger cohorts, highlighting the importance of personalized, age-specific therapeutic interventions targeting metabolic activation, suppression of apoptotic cell death, and/or modulation of the inflammatory response in patients with ischemic stroke.

*Limitations.* This study used archival autopsy samples of the cerebral cortex, which precluded assessment of infarct volume and location: only cortical tissue was identified based on pathological examination protocols and characteristic structural organization. Also, in the present study, we did not conduct a correlation analysis between the clinical data of patients and the obtained results of morphological analysis, which may be interesting and useful for future research.

## 4. Materials and Methods

Autopsy material for the study was obtained from patients (n = 154) no later than six hours after the official determination of death. Patients who died as a result of ischemic stroke were stratified into age groups according to the World Health Organization (WHO) age classification: 18–44 years—young adults (n = 38), 45–59 years—middle (n = 51), and 60–74 years—elderly (n = 65).

Archived autopsy specimens (paraffin-embedded frontal cortex blocks) were obtained from patients with a confirmed diagnosis of cerebral infarction (the International Classification of Diseases-10: I63.3/I63.4), verified by clinical and anamnestic data, neuroimaging methods (Computer tomography/Magnetic Resonance Imaging of the brain), and postmortem pathological examination. For analysis, only cases of ischemic stroke of cardioembolic or atherothrombotic origin (classified according to the Trial of ORG 10172 in Acute Stroke Treatment, TOAST) within the territories of the anterior cerebral artery (ACA) and middle cerebral artery (MCA) were included. In all patients (n = 154), the time interval from the presumed or documented onset of the disease did not exceed seven days, corresponding to the acute phase of ischemic stroke.

Exclusion criteria. Cases were excluded in the presence of cerebrovascular disorders of hemorrhagic or mixed origin of any localization or etiology; traumatic brain injury; or infarction without involvement of the frontal cerebral cortex (i.e., infarcts confined to the brainstem, cerebellum, or subcortical structures). Additional exclusion criteria included concomitant central nervous system pathology such as neurodegenerative diseases or primary/secondary brain tumors; hematological, autoimmune, and/or systemic oncological diseases; meningitis, encephalitis, or other acute bacterial and/or viral infections, including active hepatitis B or C, syphilis, or Human Immunodeficiency Virus (HIV) infection. Patients with a history of substance or alcohol abuse were also excluded. Finally, autopsy samples exhibiting tissue autolysis, insufficient material volume, or collection, fixation, and/or storage artifacts were not included in the analysis.

In all cases, informed consent was obtained from the patients’ relatives for postmortem examination and the use of biological material for research purposes. The study was approved by the Local Ethics Committee of Sechenov University (Protocol No. 13–25, dated 5 June 2025). All procedures were conducted after ethical approval was obtained.

### 4.1. Morphological Study

Serial sections (3 μm thick) were prepared from paraffin-embedded brain tissue blocks, deparaffinized, dehydrated, and stained with hematoxylin and eosin according to standard protocols. To identify neurons, one section from each case was stained with cresyl violet using the Nissl method following conventional procedures. The resulting histological slides were examined under a Leica DM2000 light microscope (Leica Microsystems, Wetzlar, Germany) equipped with a digital microphotography system. Neuronal counts were performed in 10 randomly selected microscopic fields at ×400 magnification, separately for the infarct core and the penumbra/infarct border zone. All tissue samples included in the study were confirmed to originate from the ischemic stroke region based on macroscopic and microscopic criteria. All morphological quantifications, including neuronal counts in the intact cortex, the infarct border zone, and the infarct core, were conducted by an investigator who was fully blinded to the patients’ age groups and clinical characteristics to prevent observer bias.

### 4.2. Multiplex Immunohistochemical Analysis

Multiplex immunofluorescence staining was performed using the Opal™ 7-Color Fluorescence Immunohistochemistry Kit (Akoya Biosciences, Marlborough, MA, USA) according to the manufacturer’s protocol. After deparaffinization and rehydration, antigen retrieval was carried out in Tris-EDTA buffer (pH 9.0) using a microwave oven for 15 min, followed by blocking of endogenous peroxidase activity. *First staining round*. Sections were incubated for 60 min with the primary antibody against NeuN (Clone EPR12763, Abcam, Cambridge, UK; 1:500). After washing, slides were treated with the Horseradish Peroxidase (HRP) Ms+Rb polymer for 10 min, followed by incubation with the Opal 647 fluorophore for 10 min. Bound primary and secondary antibodies were then removed by repeated microwave treatment in Tris-EDTA buffer (pH 9.0) as described above. *Subsequent staining rounds.* The procedure was repeated with antibodies against NSE (Clone EPR12483, Abcam, Cambridge, UK; 1:500) and Caspase 3 (Clone 9H19L2, Thermo Fisher Scientific, Waltham, MA, USA; 1:500). After incubation with the HRP Ms+Rb polymer for 10 min, the slides were treated with Opal 488 and Opal 540 fluorophores, each for 10 min. Nuclei were counterstained with DAPI for 5 min, and the sections were mounted using ProLong™ Diamond Antifade Mountant (Thermo Fisher Scientific, Waltham, MA, USA). *Visualization and quantitative analysis.* Multispectral images were acquired using the Vectra^®^ Polaris Automated Quantitative Pathology Imaging System (Akoya Biosciences, Marlborough, MA, USA) at 20× magnification. Whole-slide images were processed and analyzed using inForm^®^ Image Analysis Software v2.6 (Akoya Biosciences, Marlborough, MA, USA).

Then, the number of stained neurons was counted separately for the markers NeuN, NSE and caspase 3 in the field of view of a confocal microscope at a magnification of ×400. Based on the obtained average values, the percentage of Caspase 3^+^ cells among NeuN^+^ was calculated using the formula Caspase 3/NeuN ×100 (%), reflecting the proportion of dying neurons.

### 4.3. Quantitative Real-Time Polymerase Chain Reaction (qRT-PCR) Analysis

The expression levels of mRNA for proteins of PI3K/Akt/mTOR and PI3K/Akt/FOXO3a were evaluated using quantitative real-time PCR (qRT-PCR). The material for RT-PCR was collected at a distance of 1 mm from the border of the infarct core in the infarct border zone. For control, tissue was taken from a distant area of the cerebral cortex, which can be considered conditionally intact. Tissue samples were homogenized according to standard protocols. Total RNA was extracted using the *RNeasy Plus Mini Kit* (QIAGEN, Venlo, The Netherlands). Complementary DNA (cDNA) synthesis was performed using the *SuperScript™ VILO™ Master Mix* (Invitrogen, Thermo Fisher Scientific, Waltham, MA, USA).

The obtained cDNA samples were subjected to qRT-PCR using the *ABsolute™ Blue QPCR Mix* (Thermo Fisher Scientific, Waltham, MA, USA) with SYBR Green I detection dye. Amplification was carried out on the *StepOne™ Real-Time PCR System* (Applied Biosystems, Foster City, CA, USA) following the manufacturer’s instructions. Gene expression analysis was performed using the comparative threshold cycle (ΔCt) method, and relative expression levels were calculated according to the established protocol.

β-actin was used as a housekeeping reference gene for normalization which was selected based on its well-established stability in human postmortem cortical tissue. ACTB expression demonstrates minimal variability across ischemic and non-ischemic cortical regions and exhibits no significant age-dependent fluctuations, which supports its suitability for use as an internal control in qRT-PCR analysis. Furthermore, preliminary assessment of our dataset confirmed low dispersion of Ct values for β-actin across all examined samples.

Primer sequences were designed based on publicly available DNA and mRNA data from the NCBI database using the Primer-BLAST tool (Table 3).

### 4.4. Statistical Analysis

Quantitative data were processed using SPSS Statistics 12 for Windows (IBM Analytics, Armonk, NY, USA). The normality of data distribution was assessed using the Shapiro–Wilk test. For normally distributed variables, comparisons were performed using Student’s t-test or one-way ANOVA with appropriate post hoc tests. In cases of non-normal distribution, the Kruskal–Wallis test with Dunn’s post hoc correction was applied. Multiple comparisons in the clinical dataset were carried out using one-way ANOVA with post hoc analysis for normally distributed data and Pearson’s χ^2^ test for categorical variables. Pairwise comparisons in the morphological and immunohistochemical analyses were performed using the Mann–Whitney U test with Bonferroni correction to account for multiple testing. For qRT-PCR data, relative gene expression was evaluated using the ΔCt method, and intergroup differences were analyzed using Student’s t-test or one-way ANOVA with Bonferroni adjustment when applicable. The results are presented as mean ± standard deviation (SD) and, where appropriate, with a 95% confidence interval (CI). A *p*-value ≤ 0.05 was considered statistically significant.

## 5. Conclusions

Aging markedly reduces neuronal metabolic activity and increases apoptotic death in the infarct border zone after ischemic stroke. In older patients, there is an almost complete loss of NeuN and NSE expression in penumbral neurons with robust activation of the caspase cascade, whereas younger patients retain a pool of metabolically active neurons. Age-dependent dysregulation of PI3K/Akt signaling—characterized by FOXO3a hyperactivation and mTOR suppression—further promotes apoptosis and dysregulated autophagy. These changes likely underlie the limited efficacy of standard neuroprotection in ischemic stroke and support the need for age-tailored neurotropic therapy aimed at enhancing pro-survival pathways within the infarct border zone.

## Figures and Tables

**Table 1:** Average number of neurons in the zones of the infarct core, penumbra, and conditionally intact cerebral cortex one day after ischemic stroke, in one field of view.

Age	Intact Zone	Infarct Core	Penumbra Zone
18–44 y.o.	19.3 ± 0.9	4.4 ± 0.2 *	15.3 ± 0.7 *^#^
45–59 y.o.	18.8 ± 0.9	4.1 ± 0.2 *	14.7 ± 0.7 *^#^
60–74 y.o.	15.6 ± 0.7 ^!^	2.7 ± 0.1 *^!^	8.9 ± 0.4 *^#!^

Normality of distribution was assessed using the Shapiro–Wilk test, followed by Student’s *t*-test for normal distributions and the Kruskal–Wallis test with Dunn’s post hoc correction for non-normal distributions using SPSS Statistics 12 for Windows (IBM Analytics, Armonk, NY, USA). Pairwise comparisons were performed using the Mann–Whitney U test with Bonferroni correction. Statistically significant differences: *—compared with the intact zone; #—compared with the infarct core; !—compared with the “18–44 years” age group in the same localization; *p* < 0.05.

**Table 2:** Average number of NeuN^+^, NSE^+^ and Caspase 3^+^ neurons in the penumbra one day after ischemic stroke, in one field of view.

Age	NeuN^+^	NSE^+^	Caspase 3^+^	Caspase^+^ Among NeuN^+^ (%)
18–44 y.o.	11.3 ± 0.5	11.1 ± 0.5	4.8 ± 0.2	42.5%
45–59 y.o.	7.9 ± 0.3 *	7.2 ± 0.3 *	6.5 ± 0.3 *	82.3%
60–74 y.o.	4.4 ± 0.2 ^#!^	3.6 ± 0.1 ^#!^	3.9 ± 0.1 ^#!^	88.6%

Normality of distribution was assessed using the Shapiro–Wilk test, followed by Student’s *t*-test for normal distributions and the Kruskal–Wallis test with Dunn’s post hoc correction for non-normal distributions using SPSS Statistics 12 for Windows (IBM Analytics, Armonk, NY, USA). Pairwise comparisons were performed using the Mann–Whitney U test with Bonferroni correction. Statistically significant differences: *—45–59 vs. 18–44; #—60–74 vs. 18–44; !—60–74 vs. 45–59; *p* < 0.05.

**Table 3:** Primer sequences for qPCR (*Homo sapien*-specific sequences).

Gene	RefSeq	Forward Primer (5′–3′)	Reverse Primer (3′–5′)
*PIK3CA*	NM_006218	GAAGCACCTGAATAGGCAAGTCG	GAGCATCCATGAAATCTGGTCGC
*AKT2*	NM_001626	CATCCTCATGGAAGAGATCCGC	GAGGAAGAACCTGTGCTCCATG
*MTOR*	NM_004958	AGCATCGGATGCTTAGGAGTGG	CAGCCAGTCATCTTTGGAGACC
*FOXO3A*	NM_001455	TCTACGAGTGGATGGTGCGTTG	CTCTTGCCAGTTCCCTCATTCTG
*β actin (AKTB)*	NM_001101.5	CATGTACGTTGCTATCCAGGC	CTCCTTAATGTCACGCACGAT

## Data Availability

The study did not generate publicly available archival data.

## Supplementary Materials

The following supporting information can be downloaded at: https://www.mdpi.com/article/10.3390/ijms27010081/s1.
