# Supplementary material for: Aging Rewires Neuronal Metabolism, Exacerbating Cell Death After Ischemic Stroke: A Hidden Reason for the Failure of Neuroprotection
Source: Int J Mol Sci. 2025 Dec 21;27(1):81. doi: 10.3390/ijms27010081 (PMC12785814; doi:10.3390/ijms27010081)
Supplement: Supplementary file 1 [file ijms-27-00081-s001.zip › ijms-3984505-supplementary.pdf]

Supplementary Materials Table S1. Clinical characteristics of patients with IS in different age groups

**Parameter**	**M ± SD (95% CI)/n (%)**			***p***
	**Young (n = 38)**	**Middle (n = 51)**	**Elderly (n = 65)**	
**Age and gender composition of patients**				
age, years	34.8 ± 4.2 (33.42–36.18)	51.1 ± 4.8 (49.75–52.45)	69.3 ± 5.4 (67.96–70.64)	**<0.0001** [1]
male	30 (78.9%)	34 (66.7%)	47 (72.3%)	0.315 [2]
female	8 (21.1%)	17 (33.3%)	18 (27.7%)	
**Anamnestic data**				
smoking	21 (55.3%)	29 (56.9%)	38 (58.5%)	0.95
arterial hypertension	34 (89.5%)	48 (94.1%)	64 (98.5%)	0.135
hypodynamia	9 (11.6%)	17 (33.3%)	**55 (84.6%)**	**<0.0001** [2]
obesity	18 (47.3%)	29 (56.9%)	28 (43.1%)	0.140
BMI, kg/m2	21,7 [19,1 – 24,3]	25,6 [22,7 – 28,5]	22,3 [19,8 – 24,8]	0.352[3]
hyperlipidemia	22 (57.9%)	41 (80.4%)	**63 (95.4%)**	**<0.0001** [2]
Cardiomyopathies, heart defects, AF	**29 (76,3%)**	23 (45,1%)	27 (41,5%)	**0.0017**[2]
History of thrombosis (pulmonary embolism, deep vein thrombosis)	1 (2,6%)	5 (9,8%)	**19 (29,2%)**	**0.0006**[2]
Coronary heart disease	8 (21,1%)	**28 (54,9%)**	35 (53,8%)	**0.0017**[2]
Chronic heart failure	14 (36,8%)	25 (49,0%)	37 (56,9%)	0.144[2]
Diabetes mellitus	10 (26,3%)	19 (37,3%)	25 (38,5%)	0.424[2]
COPD	0	17 (33,3%)	**40 (61,5%)**	**< 0.0001**[2]
Chronic kidney disease	5 (13,2%)	9 (17,6%)	19 (29,2%)	0.115[2]
Gastric ulcer / duodenal ulcer	12 (31,6%)	21 (41,2%)	28 (43,1%)	0.496[2]
Chronic anemia	9 (23,7%)	20 (39,2%)	**32 (49,2%)**	**0.038**[2]
SARS-CoV-2 IgG+	**33 (86,8%)**	42 (82,4%)	44 (67,7%)	**0.047**[2]
Dementia / chronic cerebral ischemia	0	13 (25,5%)	**25 (38,5%)**	**< 0.0001**[2]
Taking medications:				
anticoagulants	**21 (55,3%)**	22 (43,1%)	20 (30,8%)	**0.047**[2]
antiplatelet drugs	7 (18,4%)	**25 (49,0%)**	31 (47,7%)	**0.005**[2]
antifibrinolytics	**3 (7,9%)**	1 (2,0%)	0	**0.049**[2]
**Diagnosis**				
ICD-10:				
I63.3	15 (39.5%)	28 (54.9%)	36 (55.4%)	0.164 [2]
I63.4	23 (60.5%)	23 (45.1%)	29 (44.6%)	0.173 [2]
I67.8	1 (2.6%)	13 (25.5%)	**57 (87.7%)**	**<0.0001** [2]
First diagnosed<br>Stroke in the past	37 (97,4%)<br>1 (2,6%)	48 (94,1%)<br>3 (5,9%)	59 (90,8%)<br>6 (9,2%)	0.413[2]<br>0.4132
SSS-TOAST:				
atherosclerosis	15 (39.5%)	28 (54.9%)	36 (55.4%)	0.164 [2]
cardioembolism	23 (60.5%)	23 (45.1%)	29 (44.6%)	0.173 [2]
Clinical symptoms:				
Contralateral hemiparesis	10 (26,3%)	38 (74,5%)	**52 (80,0%)**	**< 0.0001**[2]
Hemihypesthesia	11 (28,9%)	38 (74,5%)	**49 (75,4%)**	**< 0.0001**[2]

Hemianopsia	17 (44,7%)	26 (50,1%)	23 (35,4%)	0.233[2]
Motor/sensory aphasia	9 (23,7%)	39 (76,5%)	**55 (84,6%)**	**< 0.0001[2]**
Dizziness	**27 (71,0%)**	24 (47,1%)	16 (24,6%)	**< 0.0001[2]**
Headache	**19 (50,0%)**	11 (21,6%)	12 (18,5%)	**0.0013[2]**
NIHSS score at admission	10,9±2,2 (10,18 – 11,62)	17,3±2,7 (16,54 – 18,06)	**20,8±2,9 (20,08 – 21,52)**	**< 0.0001[1]**
GCS score at admission	11,6±2,5 (10,78 – 12,42)	**12,8±2,7 (12,04 – 13,56)**	9,3±2,6 (8,66 – 9,94)	**< 0.0001[1]**
Time from expected onset of symptoms to hospitalization, min	63,7±5,9 (62,24 – 65,16)	68,3±5,9 (66,64 – 69,96)	**88,1±6,6 (86,46 – 89,74)**	**< 0.0001[1]**
Thrombolysis at the prehospital stage	4 (10,5%)	12 (23,5%)	17 (26,2%)	0.159[2]
Endovascular intervention / recanalization	26 (68,4%)	32 (62,7%)	36 (55,4%)	0.405[2]
Cause of death:				
Cerebral edema with dislocation	32 (84,2%)	41 (80,4%)	49 (75,4%)	0.549[2]
Acute heart failure	4 (10,5%)	7 (13,7%)	9 (13,8%)	0.873[2]
Gastrointestinal bleeding	2 (5,3%)	1 (2,0%)	4 (6,2%)	0.418[4]
Pulmonary embolism	0	2 (3,9%)	3 (4,6%)	0.191[4]
**Time of death**				
Day 1	6 (15.8%)	**24 (47.0%)**	26 (40.0%)	**0.0073 [2]**
Day 2	9 (23.7%)	12 (23.5%)	13 (20.0%)	0.8682
Day 3	**13 (34.2%)**	6 (11.8%)	13 (20.0%)	**0.035 [2]**
Day 4	5 (13.2%)	2 (3.9%)	3 (4.6%)	0.1562
Day 5	2 (5.3%)	3 (5.9%)	6 (9.3%)	0.6867
Day 6	2 (5.3%)	1 (2.0%)	3 (4.6%)	0.6738
Day 7	1 (2.5%)	3 (5.9%)	1 (1.5%)	0.4112

For comparisons between groups using SPSS Statistics 12 for Windows (IBM Analytics, Armonk, NY, USA), the following were used: [1]—one-way ANOVA with with Tukey's post hoc; [2]—Pearson's χ^2-test, [3] – Kruskal-Wallis criterion, [4] – Fisher's exact test; statistically significant differences are shown in bold, $p \leq 0.05$. BMI – body mass index, AF – atrial fibrillation, COPD – Chronic Obstructive Pulmonary Disease, GCS – Glasgow Coma Scale
